# CircRTN4 aggravates mesangial cell dysfunction by activating the miR-513a-5p/FN axis in lupus nephritis

**DOI:** 10.1038/s41374-022-00788-6

**Published:** 2022-05-06

**Authors:** Xinyan Miao, Yuexin Tian, Lunbi Wu, Hang Zhao, Jinxi Liu, Fan Gao, Wei Zhang, Qingjuan Liu, Huifang Guo, Lin Yang, Ran Yang, Xiaojuan Feng, Shuxia Liu

**Affiliations:** 1 Department of Pathology; Center of Metabolic Diseases and Cancer Research, Institute of Medical and Health Science, Hebei Medical University; Key Laboratory of Kidney Diseases of Hebei Province, Shijiazhuang, 050017 China; 2 Department of Rheumatology, The Second Hospital of Hebei Medical University, Shijiazhuang, 050000 China; 3 Department of Nephrology, The Second Hospital of Hebei Medical University, Shijiazhuang, 050000 China; 4 Department of Pathology, Hebei Province Hospital of Chinese Medicine, Shijiazhuang, 050013 China

**Keywords:** Chronic inflammation, Lupus nephritis

## Abstract

Circular RNAs (circRNAs) are regulators of gene expression that can regulate cell proliferation and programmed cell death and serve as biomarkers in renal diseases. However, the specific traits and underlying mechanisms of circRNAs in the progression of lupus nephritis (LN) have not been elucidated. In the present study, we clarified that hsa_circ_0054595 (circRTN4) was upregulated in human renal mesangial cells (HRMCs). In cultured HRMCs, circRTN4 could enhance FN expression by directly interacting with miR-513a-5p. High circRTN4 expression in monocytes disseminated into HRMCs in an exosomal manner, thereby accelerating cell proliferation and extracellular matrix deposition. In addition, knockdown of circRTN4 in the kidney or peripheral blood alleviated renal damage in MRL/lpr and BALB/c mice. Clinically, high levels of circRTN4 were found in peripheral blood mononuclear cells and kidney tissues of LN patients, hence serving as an effective biomarker for LN detection and a novel therapeutic target. Our findings indicated that circRTN4 exacerbates mesangial cell dysfunction by activating the miR-513a-5p/FN axis in lupus nephritis.

## Abstract (web summary)

The expression of circRTN4 is significantly enhanced in lupus nephritis. Downregulation of circRTN4 expression alleviates extracellular matrix deposition by targeting the miR-513a-5p/FN axis in cultured human renal mesangial cells and pristane-induced BALB/c mice or MRL/lpr mice. In addition, monocyte circRTN4 participates in mesangial cell dysfunction in an exosome-dependent manner. Exosomal circRTN4 mzytherefore be an effective biomarker for lupus nephritis detection and a novel therapeutic target.

## Introduction

An increasing number of studies have confirmed that mesangial cell dysfunction, such as the excessive deposition of extracellular matrix (ECM), can result in glomerulosclerosis in various kidney diseases, including lupus nephritis (LN)^1–3^. Thus, understanding the mechanisms of mesangial cell dysfunction is a critical step toward the development of effective therapies for kidney diseases.

Several studies have reported crosstalk between glomerular cells and immune cells or within glomerular cells in LN progression. For instance, Lv et al.’s study found that tubular epithelial cell-derived miR-19b-3p was internalized by macrophages and led to M1 phenotype polarization, which played a critical pathological role in tubulointerstitial inflammation^4^. Interactions between CD40 L on T cells and CD40 on B cells are critical for autoantibody production in LN and further amplify the inflammatory milieu in the interstitium and glomeruli^5^. Additionally, Liu et al. indicated crosstalk between the tubule and the glomerulus in LN progression^6^. Inflammatory cell infiltration is the most important pathological change commonly seen in renal biopsies from patients with lupus nephritis^7^. Macrophages are the predominant immune cells that mediate the inflammatory process, and monocyte activation is associated with the progression of autoimmune diseases, including SLE^8^. At present, accumulating evidence has shown that the monocyte-macrophage system is involved in glomerular cell damage by secreting large amounts of cytokines, such as interleukin-1 (IL-1), interleukin-6 (IL-6) and tumor necrosis factor (TNF-α)^9,10^. However, the molecular mechanisms involved in communication between monocytes and mesangial cells have not been fully elucidated.

Exosomes, with their cargo, serve as messengers in cell-to-cell communications^11^. Proteins, metabolites, and nucleic acids delivered by exosomes can induce phenotypic and molecular alterations in recipient cells^12^. In particular, circRNAs are stably present in exosomes, thus effectively altering the biological responses of their recipient cells^13^. CircRNAs, with circular forms, represent a novel and abundant class with pivotal regulatory functions. Additionally, circRNAs are evolutionarily conserved, relatively stable, and can bind certain microRNAs (miRNAs) or RNA-binding proteins (RBPs), thus regulating gene expression at the posttranscriptional level^14–16^. Recently, deregulated expression of certain circRNAs has been identified in many types of diseases, including renal cell carcinoma and several kidney diseases^17–19^. However, whether there are differentially expressed circRNAs in exosomes released by activation of macrophages, the role of differentially expressed circRNAs in mesangial cell dysfunction, and the possible mechanisms are still unclear in the pathogenesis of lupus nephritis.

Therefore, in this study, we explored the specific roles and mechanism of deregulated circRNAs in proliferation and extracellular matrix deposition in mesangial cells and investigated whether monocytes are involved in mesangial cell injury by secreting exosomal circRNAs.

## Materials and methods

### Patients and tissue samples

Thirty patients aged 22–55 years diagnosed with SLE and type III/IV LN (ISN/RPS2003 taxonomy) were recruited from the Inpatient Department of Nephrology at the Second Hospital of Hebei Medical University from 2017 to 2019. Twenty samples of control renal tissues were obtained from nephrectomies performed for the treatment of renal lipomyoma cancers, and pathologically confirmed normal kidney tissue without a history of other autoimmune diseases, diabetic nephropathy, hypertension nephropathy, or severe infections. Peripheral blood mononuclear cell was acquired from 27 patients with active LN and 28 patients of SLE without kidney damage. All patients met the 2012 revised criteria for SLE and LN and were diagnosed at the Department of Rheumatology of the Second Hospital of Hebei Medical University from August 2017 to June 2019. Plasma samples for cell culture were acquired from five patients of LN without the initiation of immunosuppressive therapy, infections or other complications who underwent therapeutic plasma exchange. Five plasma samples were collected from healthy donors whose age and sex-matched the LN patients (UPro = 2.186 ± 0.96 g/24 h) and was named the control group. The existing exosomes in these plasma samples were removed before incubating with cells. This study was approved by the Clinical Research Ethics Committee of the Second Hospital of Hebei Medical University. Written informed consent was obtained from each study participant.

### Animals

We generated two mouse models: (1) Female MRL/lpr mice and MRL/MPJ mice were purchased from Jackson Laboratory (#JAS000485, Bar Harbor, ME, USA, RRID: IMSR_JAX: 000485). (2) A pristane-induced lupus model was generated with BALB/c mice (HFK Bioscience Co., Ltd, Beijing, China). According to the article^20^, disease induction is performed through an intraperitoneal injection of 0.5 ml of pristane. After six months, dsDNA level in peripheral blood was detected to identify whether the model was successfully established (BALB/c + P). IgG expression in the kidney was detected by IF and urine protein level was detected by ELISA to identify the renal injury.

Mice were fed in a standard environment with regular light/dark cycles and free access to a water and food diet. All animal experiments were approved by the Institutional Animal Care and Use Committee of Hebei Medical University (approval ID: HebMU 20080026). BALB/c + P mice were randomly divided into five groups: BALB/c + P, BALB/c + P + CircRTN4-shRNA-AAV (BALB/c + P + CircRTN4-R) or BALB/c + P + circNC-AAV (BALB/c + P + circNC-R) by kidney orthotopic injection and BALB/c + P + CircRTN4-shRNA-AAV (BALB/c + P + CircRTN4-V) or BALB/c + P + circNC-AAV (BALB/c + P + circNC-V) group by tail vein injection. Six BALB/c mice served as the control group. Similarly, MRL/lpr mice were randomly divided into five groups: MRL/lpr, MRL/lpr + CircRTN4-shRNA-AAV (MRL/lpr + CircRTN4-R) or MRL/lpr + circNC-AAV (MRL/lpr + circNC-R) by kidney orthotopic injection and MRL/lpr + CircRTN4-shRNA-AAV (MRL/lpr + CircRTN4-V) or MRL/lpr + circNC-AAV (MRL/lpr + circNC-V) group by tail vein injection. Three MRL/MPJ mice served as the control group.The adeno-associated virus (AAV) contained specific sicircRTN4 target or negative control target was purchased from VectorBuilder (Guangzhou, China)). For kidney orthotopic injection: the renal cortex of mice in the CircRTN4-R group and the NC-R group were injected with 50 μL 1 × 10^9^ infective units of AAV in both kidneys, while the control BALB/c or MRL/MPJ mice were injected with isometric saline. For tail vein injection: we administrated 100 μL 1 × 10^9^ infective units of AAV via tail vein in CircRTN4-V and circNC-V groups, while the control BALB/c or MRL/MPJ mice were injected with isometric saline. After six weeks, the mice were sacrificed after 24-h urine and blood samples were taken, and the renal cortex was collected for relevant investigations. The 24 h proteinuria was detected by a mouse urine protein enzyme-linked immunosorbent assay quantification kit according to the manufacturer’s protocol (ZCi Bio, Shanghai, China, #ZC-38527). Blood urea nitrogen (BUN) and serum creatinine (Scr) were analyzed by the Urea Assay Kit (Jiancheng Bio, Nanjing, China, #C013-2-1) and Creatinine Assay Kit (Jiancheng Bio, Nanjing, China, #C011-1-1) according to the manufacturer’s protocol.

### Cell culture

HRMCs and the THP1 cell line were purchased from the Chinese Academy of Sciences, Shanghai Institute for Biological Sciences Cell Resource Center. HRMCs and THP1 cells were cultured in Roswell Park Memorial Institute (RPMI) 1640 medium with 10% exosome-free fetal bovine serum (FBS) (Gibco, NY, USA). HEK293T cells were cultured in Dulbecco’s modified Eagle’s medium (DMEM) with 10% FBS (Gibco, NY, USA). Culture conditions were carried out at 37 °C in an incubator (Thermo Fisher Scientific, Waltham, MA, USA) with 5% CO_2_ and 95% air. (1) To investigate the effect of LN plasma on the expression of circRTN4, the HRMCs and THP1 were randomly exposed to control plasma or LN plasma (5%) and collected at 8 h. Real-time PCR was used to examine the expression of circRTN4. (2) In the RNA interference (RNAi) experiment, the HRMCs were randomly divided into four groups: control, LN plasma, LN plasma + sicircRTN4, and LN plasma + siNC. Cells were collected at different times after the stimulation with LN plasma (5%). Flow cytometry and EdU assay were used to detect the proliferation level. Then, the expression of FN was detected by real-time PCR and western blot. (3) To explore the communication between HRMCs and THP1, we employed a coculture system. THP1 cells were treated with LN plasma for 8 h, and then the culture medium was changed to an exosome-free medium for 24 h. By this way, LN plasma treated conditioned media (LM) from THP1 cells was harvested for co-culture system. HRMCs were randomly divided into four groups: control, conditioned media (LM), LM^GW4869^, LM^DMSO^. The LM^GW4869^ and LM^DMSO^ groups were pretreated with GW4869 (20 μM) or DMSO (20 μM) for 2 h. EdU assay was used to detect the proliferation level. Then, the expression of FN in HRMC and culture supernatant was detected by real-time PCR and ELISA, respectively. (4) To investigate the effect of THP1-exosome on mesangial cells, exosomes from THP1 were isolated after incubated with LN plasma or control plasma for 8 h to treat HRMCs. HRMCs were randomly divided into four groups: control, LN plasma, LN plasma + THP1exo^LN^, LN plasma + THP1exo^Con^. The expression of circRTN4 was detected by real-time PCR. (5) To investigate the effect of exosomal-circRTN4 on mesangial cells, THP1 was infected with sicircRTN4 or siNC for 48 h followed by LN plasma treatment for 8 h. THP1 derived exosomes was harvested to incubate with HRMCs. HRMCs were randomly divided into four groups: control, LN plasma, LN plasma + THP1exo^LN^, LN plasma + THP1exo^sicircRTN4+LN^, LN plasma + THP1exo^siNC+LN^. The expression of circRTN4 was detected by real-time PCR.

### Cell transfection

HRMCs and THP1 were transfected with sicircRTN4 (RIBOBIO, Guangzhou, China), and HRMCs were also transfected with circRTN4-WT (General BIO, Anhui, China) or miRNA mimics (RIBOBIO, Guangzhou, China) using Lipofectamine 3000 according to the manufacturer’s protocols (Invitrogen, Carlsbad, CA, USA, #L3000015). The sequences are as follows: sicircRTN4 (5′-TCT GAA GAT GAG ACC CTT T-3′); miR-513a-5p mimic (5′-UAC UGU GGA GGG ACA CUU-3′); miR-607 mimic (5′-GUU CAA AUC CAG AUC UAU AAC-3′); miR-647 mimic (5′-GUG GCU GCA CUC ACU UCC UUC-3′).

### Real-time PCR

Total RNA of cells and renal cortex was extracted using TRIzol reagent (Invitrogen, Carlsbad, CA, USA). RNA concentration was measured by NanoDrop 2000. Reverse transcription was performed by PrimeScript™ RT Master Mix (Takara, #047A), and polymerase chain reaction (qPCR) was performed with SYBR Premix EX Taq II (Takara, #820A) following the manufacturer’s instructions. The 2^−ΔΔCT^ method was used to normalize the qPCR cDNAs. All experiments were repeated at least in triplicate.

### RNA fluorescence in situ hybridization (FISH)

A specific probe for circRTN4 was designed and synthesized by RiboBio, and the signals were detected by the FISH Kit (RiboBio) according to the manufacturer’s instructions. Cells and tissue samples were fixed in 4% formaldehyde for 10 min. After permeabilization (1× PBS/0.3% Triton X-100), they were hybridized in hybridization buffer with specific probes to circRTN4, U6 and 18S at 37 °C overnight. The hybridization buffer was then gradually washed off with 4× SSC (including 0.1% Tween-20), 2× SSC and 1× SSC at 42 °C. Nuclei were counterstained with 4,6-diamidino-2-phenylindole (DAPI; Southernbiotech, Birmingham). Confocal images were captured using a laser scanning confocal microscope (Leica, Wetzlar, Germany). The positive signal was located in nuclei or cytoplasm and the average integrated optical density (IOD) value was quantified to indicate the expression of circRTN4.

### Immunofluorescence (IF)

4-μm-thick sections were dewaxed in xylene and rehydrated with graded ethanol. The slides were blocked with 10% goat serum and then incubated with anti-FN (1:200; Abcam, #ab2413) and anti-CD68 (1:100; ABclonal, #A13286) antibodies at 4 °C overnight. The next day, the sections were incubated with FITC-conjugated goat anti-rabbit IgG/TRITC-conjugated goat anti-mouse IgG at 37 °C for 2 h followed by treatment with 4’,6-diamidino2-phenylindole (DAPI; Southernbiotech, Birmingham). Images of the sections were captured with a laser scanning confocal microscope (Leica, Wetzlar, Germany). Image Pro Plus (Media Cybernetics, Silver Spring, MD) was used to quantify the results, and the average IOD value was quantified to indicate the expression of protein.

### Flow cytometry (FCM)

When transfected siRNA with or treated with conditioned media for 48 h, HRMCs were collected, washed with PBS, and then fixed with 75% ethyl alcohol at 4 °C overnight. The following day, the ethylalcohol was removed, and the cells were washed with PBS two times, then fixed with 500 µl of propidium iodide (PI) staining solution and incubated for 30 min in the dark at 4 °C. The analysis of the cell cycle was performed with a FACSCalibur flow cytometer with CellQuest software (Beckman).

### Immunofluorescence (IF) for EdU incorporation

The HRMC proliferation level was evaluated by measuring the incorporation of EdU using the EdU Cell Proliferation Kit (Beyotime Biotechnology, Shanghai, China) according to the manufacturer’s instructions. Firstly, the cells were treated as described above. Then, EdU (10 μmol/l) was added to the culture medium 2 h before the cells were collected. Secondly, the cells were fixed in a 4% paraformaldehyde solution and permeabilized with 0.3% Triton X‐100 for 30 min at room temperature. After that, the cells were incubated with click reaction solution for 30 min at room temperature. After washed with PBS, they were incubated with 4′,6‐diamidino‐2‐ phenylindole (DAPI; Southernbiotech, Birmingham), and the cells were observed using an Olympus microscope (OLYMPUS, Tokyo, Japan). The positive signal was located in nuclei and green granular, and the positive ratio was quantified by Image Pro Plus (Media Cybernetics, Silver Spring, MD).

### Immunohistochemistry (IHC)

The 4% formaldehyde-fixed renal sections were deparaffinized in xylene and rehydrated through graded ethanol. After antigen recovery was performed using a pressure cooker, the endogenous peroxidase was blocked with 3% H_2_O_2_ for 30 min at room temperature. Then the slides were blocked with 10% goat serum and were respectively incubated with primary antibodies against FN (1:200; Abcam, #ab2413) and CyclinD1 (1:200; Abcam, #ab134175) overnight at 4 °C. Then the sections were incubated with polymer helper and polyperoxidaseanti-mouse/rabbit IgG at 37 °C and finally stained with diaminobenzidine. Finally, images were captured using an Olympus microscope (OLYMPUS, BX71, Tokyo, Japan). The average integrated optical density value was quantified to indicate the expression of protein by Image Pro Plus (Media Cybernetics).

### Western blot analysis

Total protein from HRMCs, THP1 cells or the renal cortex of mice was extracted with RIPA lysis buffer, lysed for 1 h, and then centrifuged at 120,000 × *g* at 4 °C for 20 min. The supernatant was collected, and the relative levels of protein expression were detected by western blotting. In brief, the protein was separated by 10% sodium dodecyl sulfate-polyacrylamide gel electrophoresis (SDS-PAGE) and transferred to a polyvinylidene fluoride (PVDF) membrane (Millipore, Billerica, USA). Then the membrane was blocked with 5% bovine serum albumin (BSA) for 1.5 h at 37 °C and incubated with anti-FN (1:2000; Abcam, ab2413), CyclinD1 (1:1000; Abcam, ab134175), CD9 (1:200; Santa Cruz, sc13118), and CD63 (1:200; Santa Cruz, sc5275) antibodies at 4 °C overnight. Subsequently, the membrane was incubated with a goat anti-rabbit/mouse IgG secondary antibody (Proteintech, Wuhan, China, diluted 1:5000). Finally, the signal was imaged using the LI-COR Odyssey Infrared Imaging System (Lincoln, NE, USA). All experiments were repeated at least three times.

### Dual luciferase reporter assay

HEK-293T cells were seeded in 24-well plates at a density of 6 × 10^4^ cells per well for 24 h before transfection. The cells were co-transfected with a mixture of luciferase reporter vectors (pmirGLO) containing circRTN4-miR-513 binding sequences or mutant sequences and miRNA mimics (20 nM) to examine miRNA binding ability. After 24 h, the luciferase activity was measured using a dual luciferase reporter assay system (Promega, Madison, WI, USA) according to the manufacturer’s protocol.

### Hematoxylin-Eosin (H&E) staining

2-μm paraffin sections were deparaffinized and placed in hematoxylin solution for 5 min to stain the nuclei. Then transfer the slides to a staining jar with running water (tap water is fine) till the water is clear. After that, the slides were incubated with Eosin solution for 2 min to stain the cytoplasm. Successively transfer the slides into staining jars with 85% ethanol for 3 s, 95% ethanol for 2 min, 100% ethanol for 10 min and xylene for 10 min. Finally, the number of glomerular cells was quantitatively analyzed using Image J. Refer to the method in article^21^, an average of 30 glomeruli per mouse was examined and averaged for revealing the level of glomerular cell proliferation.

### Periodic Acid-Schiff (PAS) staining

After deparaffinized and rehydrated, the 2-μm paraffin sections were stained with a PAS staining kit (Baso, #BA4080A). Light microscopy (OLYMPUS, BX71) was used to observe morphological changes in the glomeruli. The mesangial matrix was identified by the presence of PAS-positive and nuclei-free areas in the mesangium. The intensities of these areas were quantitatively analyzed using Image J. An average of 30 glomeruli per mouse was examined and averaged for morphometric scores^21^.

### Sirius red/fast green collagen staining

After deparaffinized and rehydrated, the 4‐μm sections were stained with Dye Solution for 30 min and then eluted with Dye Extraction solution (Chondrex, # 9046). Samples for staining were dehydrated in 100% ethanol, washed with xylene and mounted in the resinous medium. Finally, the collagen was identified by the presence of Sirius Red-positive areas in the glomeruli and analyzed using Image J. An average of 30 glomeruli per mouse was examined and averaged for revealing the level of ECM deposition.

### Exosome experiments

For exosome extraction, the cells were cultured in a medium with exosome-free FBS, which was prepared by centrifugation to remove existing exosomes. For cell incubation, we followed the standard centrifugation steps. Briefly, centrifugation was performed at 300 × *g* for 10 min, followed by 2000 × *g* for 20 min and 10,000 × *g* for 30 min. The supernatant was then filtered through a 0.2-μM filter (Pall Corp) and further centrifuged at 100,000 × *g* for 90 min at 4 °C twice to pellet the exosomes. Finally, exosomes were resuspended in PBS (usually 50 μL to 100 μL). Samples were detected by transmission electron microscopy (TEM) to analyze the diameter and shape. For RT-qPCR or western blot detection, exosomes from cell culture media were isolated by the Total Exosome Isolation Reagent (from cell culture media) (Cat# 4478359, Invitrogen, USA) and exosomes from serum of mice were isolated by Total Exosome Isolation Kit (from plasma) (Cat#4484450, Invitrogen, USA), following the manufacturer’s protocol.

### Extraction of peripheral blood mononuclear cell (PBMC)

5 mL peripheral venous blood from LN patients or healthy examiner and 2 mL from mice were collected, and Human/Mouse Mononuclear Cells Separation Medium (HuahuiBio, #25610/25617) was used to extract PBMC. Cells were cultured with 1640 medium and 10% fetal bovine serum (Gibco). 24 h later, the medium supernatant was changed with fresh medium, and the CD45^+^ adherent cells were identified as monocyte.

### Enzyme-linked immunosorbent assay (ELISA)

FN (Shanghai Zcibio Technology, ZC35077; ZC38792) was analyzed using ELISA kits according to the manufacturer’s protocols. The absorbance at 450 nm was immediately measured by an automatic microplate reader after adding the stop solution.

### Statistical analysis

The data are expressed as the mean ± standard error of the mean (SEM) or median with interquartile range. SPSS 21.0 (SPSS, Inc., Chicago, IL) was used for data analysis. The homogeneity variance was compared in every group. Student’s *t* test and Mann–Whitney nonparametric test were applied to compare the variables between the two groups. One-way analysis of variance (ANOVA) was performed to evaluate the statistical significance between multiple comparisons by Bonferroni’s correction. A *P* value < 0.05 was considered statistically significant.

## Results

### CircRTN4 was high-expressed in mesangial cells of LN

**Fig. 1:**
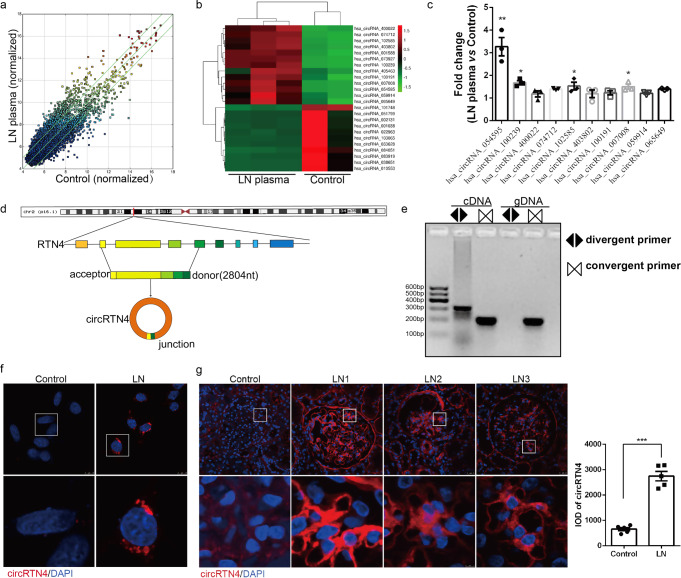
CircRTN4 is highly expressed in mesangial cells exposed to LN plasma. **a** Scatter plot comparing the expression fold changes of circRNAs in mesangial cells of LN versus the control group. The green lines indicate fold change. **b** Cluster heatmap showing differentially expressed circRNAs in HRMCs according to microarray results. **c** Detection of 10 upregulated circRNAs in HRMCs by RT-qPCR, normalized to 18S. **d** Schematic illustration of circRTN4. **e** Identification of circRTN4 PCR amplification products by DNA gel electrophoresis. **f** RNA FISH showing the predominant cytoplasmic distribution of circRTN4 in HRMCs. **g** The localization of circRTN4 in kidney tissues was detected by RNA FISH. ^*^P < 0.05, ^**^P < 0.01 and ^***^P < 0.001, One-way ANOVA followed by Bonferroni’s test. Data are presented as means ± SEM.

To explore whether deregulated circRNAs were involved in mesangial cell injury in LN, HRMCs treated with LN plasma or control plasma were characterized by an Arraystar Human CircRNA Array (Kangcheng Biotechnology Co., Ltd.) (Fig. 1a). Thirteen of the most upregulated and ten of the most downregulated circRNAs are displayed in the heatmap (Fig. 1b). By RT-qPCR, we identified 10 of the most deregulated circRNAs. As shown in Fig. 1c, the hsa_circ_0054595 was notably upregulated in HRMCs exposed to LN plasma. As the results showed in circBase, hsa_circ_0054595 consisted of RTN4 exons 4–8, referred to as circRTN4 hereafter (Fig. 1d). To further confirm the existence and circular characteristics of circRTN4, we performed qPCR using divergent primers containing a back-splice junction and convergent primers for both cDNA and genomic DNA (gDNA) and discovered that divergent primers amplified only a band in cDNA (Fig. 1e). The predominant cytoplasmic distribution of circRTN4 in HRMCs was revealed by FISH staining (Fig. 1f). Moreover, we validated the expression of circRTN4 in clinical specimens. As shown in Fig. 1g, the positive staining of circRTN4 was located in cytoplasm in glomerular cells and significantly higher in LN patients than that in normal group, and the positive position of circRTN4 was consistent with the mesangial area. Collectively, these data indicated that circRTN4 expression is upregulated in both HRMCs and human kidneys of LN.

### Downregulation of circRTN4 suppressed the proliferation and FN expression of HRMCs induced by LN plasma

**Fig. 2:**
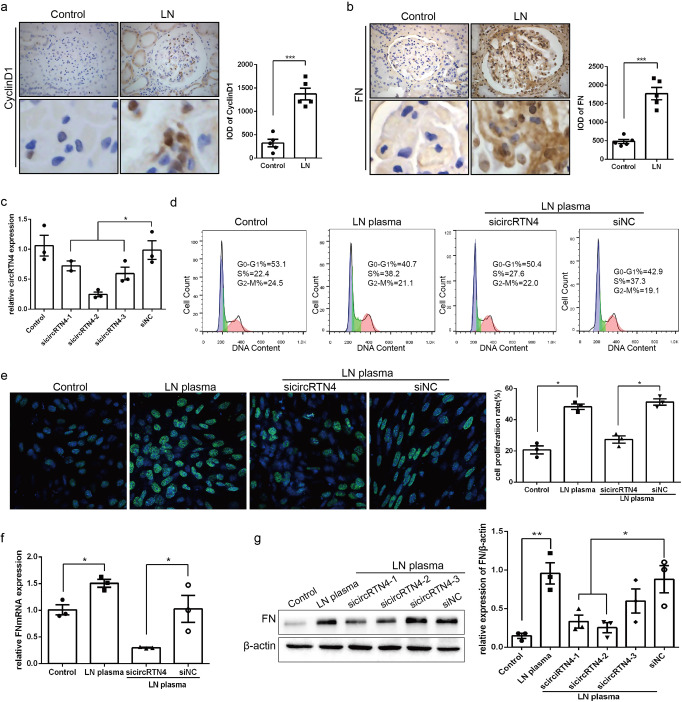
CircRTN4 knockdown affects cell proliferation and FN expression in HRMCs with LN plasma treatment. **a**, **b** Immunohistochemistry and quantitative analysis of CyclinD1 and FN protein expression in the glomeruli of LN patients. ^***^P < 0.001, Student’s *t* test. **c** CircRTN4 knockdown efficiency was analyzed by RT-qPCR. **d**, **e** FCM and EdU assays showed that circRTN4‐siRNA transfection suppressed the proliferation level of HRMCs. **f**, **g** The expression of FN was detected by RT-qPCR and Western blot. **c**, **f**, **g** ^*^P < 0.05 and ^**^P < 0.01, One-way ANOVA followed by Bonferroni’s test. Data are presented as means ± SEM.

Our previous study proved that mesangial cell dysfunction plays an important role in LN progression through excessive cell proliferation and accumulation of extracellular matrix (ECM)^22^. Consistent with previous experimental results, we confirmed again that CyclinD1 and FN were highly expressed in the glomeruli of LN patients (Fig. 2a, b). To further explore the role of circRTN4, siRNA was designed targeting the junction site of circRTN4. As shown by RT‐qPCR, the expression of circRTN4 was reduced in the circRTN4‐specific siRNA group compared with that in the negative control siRNA group (Fig. 2c) and we selected the sicircRTN4-2 for follow-up research. As illustrated in Fig. 2d, the results of Flow cytometry showed the percentage of cells in G_0_/G_1_ phage was remarkably decreased accompanied with S phrase increased in the LN group, whereas knockdown of circRTN4 expression inhibited the effect of LN plasma. Similarly, the EdU assay revealed that downregulation of circRTN4 impaired the excessive proliferation of HRMCs incubated with LN plasma (Fig. 2e). Moreover, we found that FN mRNA level showed a remarkable decrease with the downregulation of circRTN4 compared with the LN group (Fig. 2f), which showed the same change in the level of FN protein (Fig. 2g). In brief, downregulation of circRTN4 likely suppressed the proliferation and FN production of HRMCs in LN group.

### CircRTN4 enhanced FN expression by directly interacting with miR-513a-5p

**Fig. 3:**
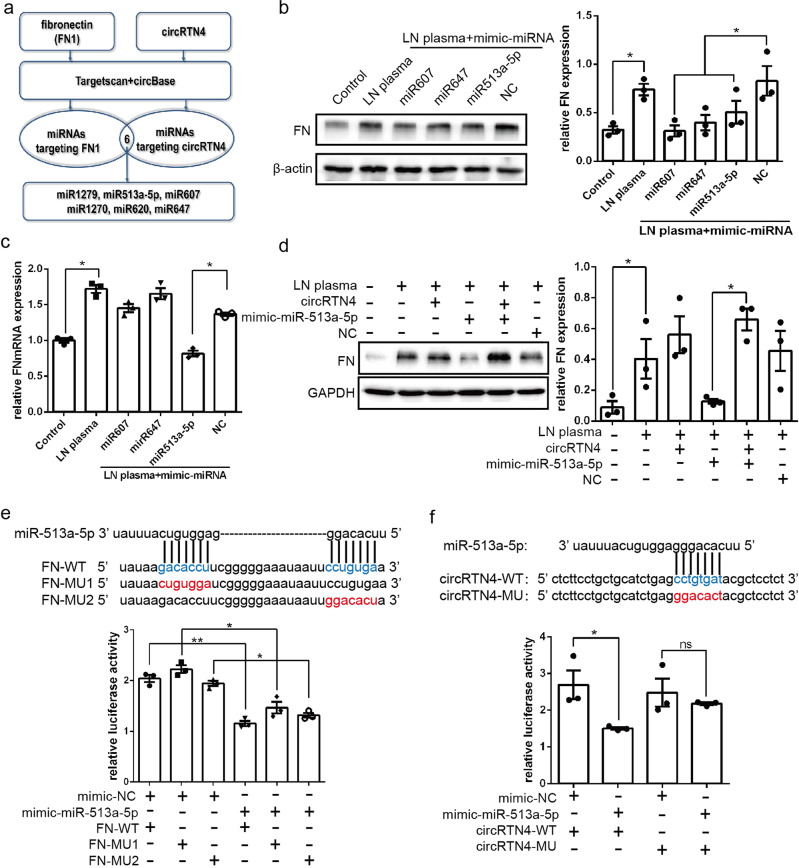
CircRTN4 enhanced FN expression by sponging miR-513a-5p. **a** The schematic flowchart shows the miRNAs that could bind to circRTN4 and the FN 3′-UTR via an online bioinformatic network. **b**, **c** FN expression in HRMCs detected by Western blot and RT-qPCR. **d** Western blotting analysis showed that miR-513a-5p could decrease the protein level of FN and that the addition of circRTN4 could reverse this effect in HRMCs. **e**, **f** A dual-luciferase reporter assay was performed to confirm the binding site of miR-513a-5p, FN and circRTN4. ^*^P < 0.05 and ^**^P < 0.01, One-way ANOVA followed by Bonferroni’s test. Data are presented as means ± SEM.

It has been well demonstrated that the function of circRNA is closely related to its location. Previously, we confirmed the predominant cytoplasmic distribution of circRTN4 not only in HRMCs but also in renal glomerular of LN group. Then, we speculated that circRTN4 might act as a molecular sponge for miRNAs to regulate downstream genes. To validate this hypothesis, online bioinformatics database analysis (CircInteractome and miRDB) identified that six miRNAs could target FN mRNA while sponging by circRTN4 (Fig. 3a). Since it was the result of miRDB prediction, the higher the score, the higher the reliability. Results with a score greater than 80 are generally considered relatively reliable, while that below than 60 are considered less reliable. Three miRNAs (miR-607, miR-647, miR-513a-5p) with higher target scores (greater than 75) were selected for further investigation. And these three miRNAs could all reduce the expression of FN protein (Fig. 3b). Whereas only miR-513a-5p could downregulate FN mRNA level (Fig. 3c), thus we hypothesized that miR-513a-5p could directly bind with the FN 3’-UTR sequence, while the other two miRNAs might indirectly regulate the expression of FN. Moreover, this effect could be reversed after transfection with the circRTN4 overexpression plasmid (Fig. 3d). Next, to determine whether miR-513a-5p could bind to FN mRNA directly, we constructed a reporter plasmid with the wild-type FN 3′-UTR and the 3′-UTR with potential site mutations. As anticipated, miR-513a-5p mimics dramatically reduced the luciferase activity of 293T cells transfected with the plasmid containing the wild-type FN mRNA 3′-UTR sequence (Fig. 3e). The dual luciferase reporter vector also confirmed the interaction and the binding site of miR-513a-5p and circRTN4 in 293T cells (Fig. 3f). These data indicated that by sponging miR-513a-5p, circRTN4 alleviated the overexpression of FN induced by LN plasma.

### Downregulation of kidney circRTN4 inhibited ECM accumulation in LN mice

**Fig. 4:**
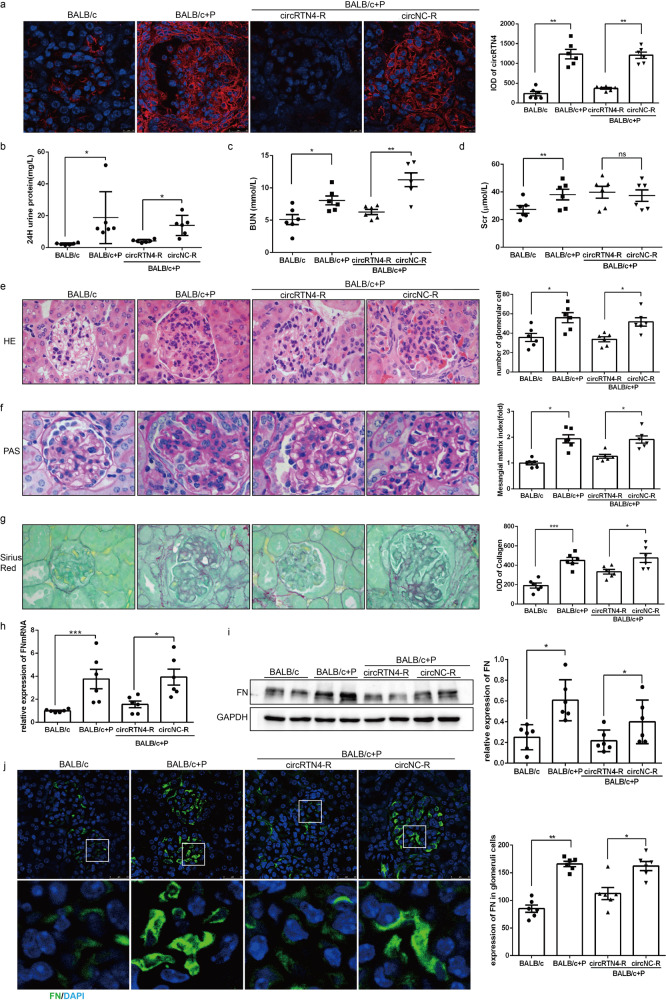
Downregulation of kidney circRTN4 inhibited ECM accumulation in LN mice. **a** RNA FISH showed the expression and location of circRTN4 in LN mice. **b** Twenty-four-hour proteinuria levels were detected by ELISA. **c**, **d** Level of BUN and Scr in LN mice. **e** H&E staining of kidney and analysis of cell number of glomeruli. **f** PAS staining and semiquantitative analysis of mesangial expansion and extracellular matrix deposition. **g** Sirius red staining and semiquantitative analysis of collagen in glomerular. **h** RT-qPCR assays for FN mRNA levels in the renal cortex. **i** Western blot analysis and quantitative analysis of FN protein levels in the renal cortex. **j** IF staining and quantitative analysis of FN protein levels in the glomeruli of LN mice. *n* = 6 for each group. ^*^P < 0.05, ^**^P < 0.01 and ^***^P < 0.001, One-way ANOVA followed by Bonferroni’s test. Data are presented as means ± SEM.

To further confirm the role of circRTN4 in the dysfunction of mesangial cells in *vivo*, we used two models including pristane-induced LN model and MRL/lpr mice. In pristane-induced LN model, the expression of circRTN4 was increased in glomerular cells of BALB/c + P mice and located in cytoplasm, while the expression of circRTN4 was notably reduced in the BALB/c + P + circRTN4-R group compared with the BALB/c + P group (Fig. 4a). In addition, knockdown of circRTN4 expression in glomerular cells alleviated proteinuria and blood urea nitrogen (BUN) levels in the BALB/c + P + circRTN4-R group (Fig. 4b, c), while serum creatinine (Scr) remained unchanged (Fig. 4d). H&E, PAS and Sirius Red staining revealed that the cell proliferation, mesangial expansion and matrix accumulation in the glomeruli of BALB/c + P mice were rescued by injection of circRTN4‐shRNA‐AAV (Fig. 4e–g). The mRNA and protein levels of FN were significantly increased in BALB/c + P group compared with control group, whereas FN expression was markedly reduced after downregulation of circRTN4 (Fig. 4h–j). Similar results were shown in MRL/lpr mice (Supplementary Fig S1). In general, inhibition of circRTN4 in glomeruli could alleviate the pathological changes and improve the renal function of LN mice.

### Increasing internalization of monocyte-derived exosomes aggravated HRMC cell proliferation and FN expression

**Fig. 5:**
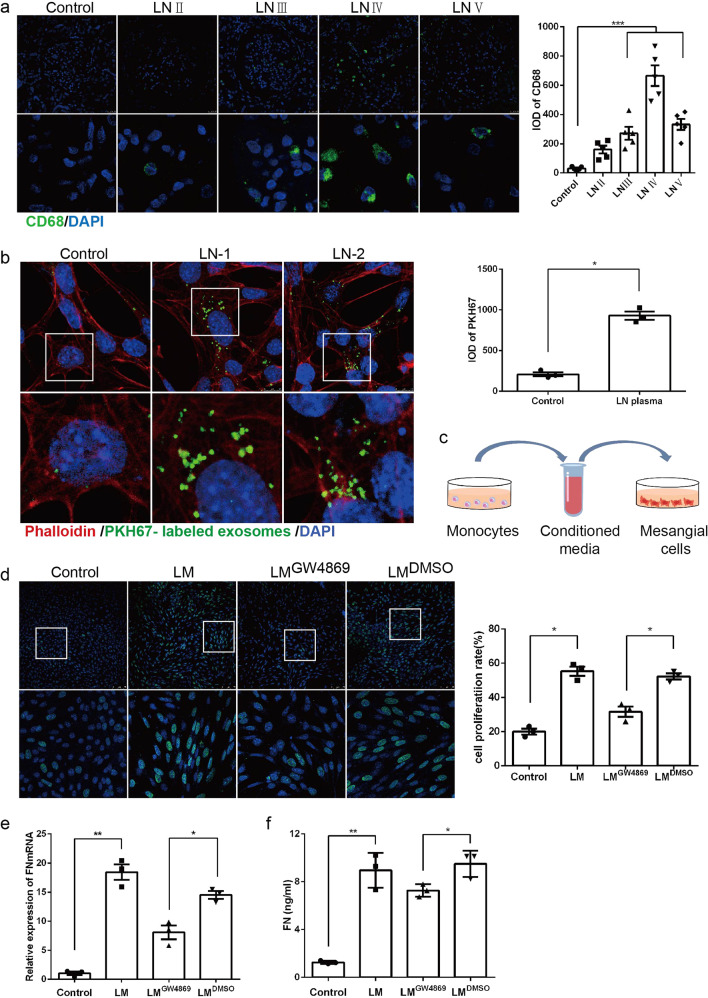
Monocyte-derived exosomes accepted by HRMCs aggravated mesangial cell dysfunction. **a** IF and quantitative analysis of CD68 confirmed macrophage infiltration in the glomeruli of LN patients. **b** PKH67-labeled exosomes were applied to recipient HRMCs, and fluorescence was captured with a confocal microscope. **c** The co-culture system of THP1 and HRMCs. **d** HRMCs were incubated with LM for 8 h, and EdU assay showed that inhibition of exosomes suppressed the proliferation level of HRMCs. **e** HRMCs were incubated with LM for 48 h, and the expression of FN was detected by RT-qPCR. **f** HRMCs were incubated with LM for 48 h, and the expression of FN in culture supernatant was detected by ELISA. ^*^P < 0.05, ^**^P < 0.01 and ^***^P < 0.001, One-way ANOVA followed by Bonferroni’s test. Data are presented as means ± SEM.

Monocytes/macrophages participate in inflammatory damage to local tissues and organs. Unsurprisingly, we confirmed marked macrophage infiltration of LN kidney tissues (Fig. 5a). To further investigate whether exosomes were involved in the intercellular communication of LN, we isolated and purified PKH67-labeled monocyte-derived exosomes by ultracentrifugation and applied them to HRMCs. As expected, increased internalization of fluorescent exosomes was observed in HRMCs treated with LN plasma (Fig. 5b). To further confirm the direct effect of exosomes between cells, we employed a co-culture system (Fig. 5c). THP1 cells were treated with LN plasma for 8 h, and then the culture medium was changed to an exosome-free medium. Twenty-four hours later, conditioned media (LM) from THP1 cells was harvested to treat HRMCs. As shown in EdU assays, LM from LN plasma-treated THP1 cells significantly promoted HRMC proliferation, whereas blocking exosome release by GW4869 in THP1 cells could alleviate the excessive proliferation of HRMCs (Fig. 5d). Consistent with this, the overexpression and secretion of FN induced by LM were remarkably reduced by the addition of GW4869 (Fig. 5e, f). Taken together, monocyte-derived exosomes were transported to HRMCs, thus promoted cell proliferation, FN synthesis and secretion.

### Monocyte-circRTN4 diffused into HRMCs, thus increased its expression in HRMCs

**Fig. 6:**
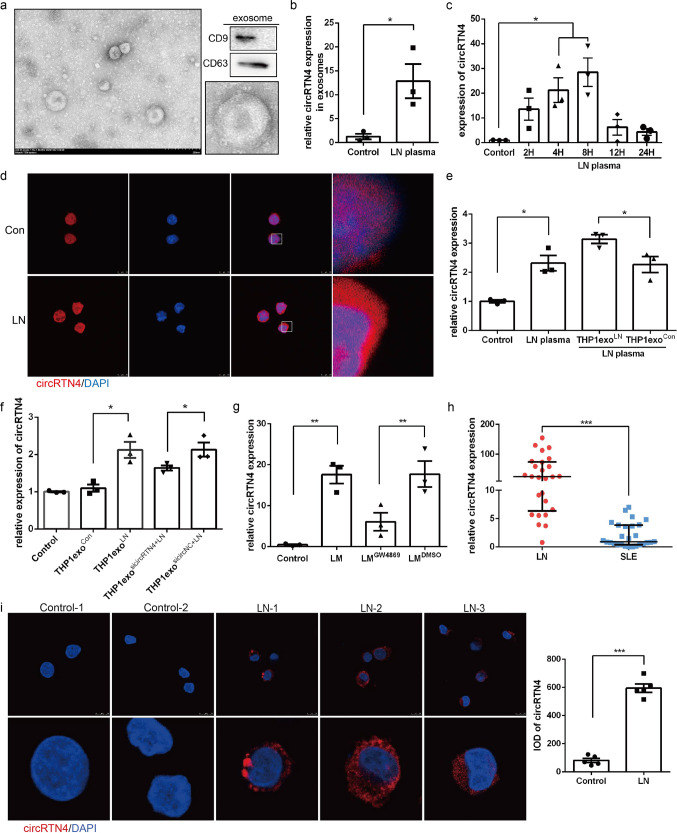
High expression of circRTN4 in monocytes diffused into recipient HRMCs. **a** Exosome identification by TEM and Western blot analysis of the expression of the exosome marker proteins CD9 and CD63. **b** The expression of circRTN4 in THP1 exosomes from culture supernatant. ^*^P < 0.05, Student’s *t* test. **c** RT-qPCR showed circRTN4 expression in THP1 cells cultured with 5% LN plasma. **d** The localization of circRTN4 in THP1 cells was detected by RNA FISH. **e** RT-qPCR showed circRTN4 expression in HRMCs cultured with THP1 exosomes and 5% LN plasma for 8 h. **f** The expression of circRTN4 in HRMCs incubated with exosomes derived from THP1 cells for 8 h. **g** HRMCs were incubated with LM for 8 h, and the expression of circRTN4 was detected by RT-qPCR. **h**, **i** Detection of circRTN4 in peripheral blood mononuclear cells (PBMCs) by RT-qPCR (LN: *n* = 26, SLE: *n* = 27) and RNA FISH (*n* = 5). **h** ^***^P < 0.001, Mann–Whitney test. Data are presented as median with interquartile range; **c**, **e**, **f**, **g**, **h** ^*^P < 0.05 and ^**^P < 0.01, One-way ANOVA followed by Bonferroni’s test. Data are presented as means ± SEM.

To further investigate the specific mechanism of exosome in intercellular communication between cells, THP1-derived exosomes were extracted and identified by TEM and western blot (Fig. 6a). And we demonstrated that circRTN4 was significantly enriched in exosomes and THP1 cells incubated with LN plasma (Fig. 6b, c). FISH revealed the predominant cytoplasmic localization of circRTN4 in THP1 cells (Fig. 6d). Subsequently, HRMCs were incubated with exosomes isolated from THP1 cells. As shown in Fig. 6e, LN plasma-treated THP1 exosomes further elevated the high expression of circRTN4 in HRMCs induced by LN plasma. Meanwhile, the level of circRTN4 in exosome-exposed HRMCs was obviously decreased by knockdown the circRTN4 expression in THP1 cells (Fig. 6f). Then, the inhibition of exosomes reduced the expression of circRTN4 in HRMCs treated with LM (Fig. 6g). The above results indicated that upregulated exosomal-circRTN4 could induce dysfunction of mesangial cells. Importantly, total RNA was extracted from PBMCs of SLE patients without kidney damage and LN patients, the expression of circRTN4 was significantly enriched in LN patients (Fig. 6h). RNA FISH revealed the predominant cytoplasmic localization of circRTN4 in PMBCs (Fig. 6i). In summary, THP1 derived exosomal circRTN4 might play a vital role in LN progression through interacting with HRMCs.

### Downregulation of circRTN4 in PBMCs alleviated renal damage in LN mice

**Fig. 7:**
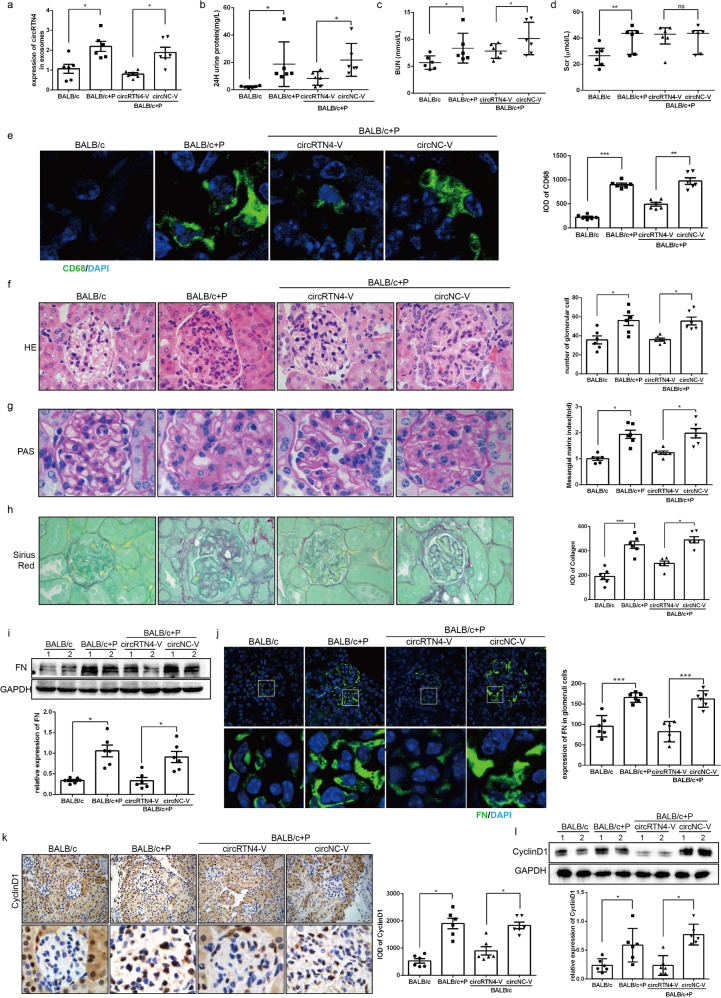
Downregulation of peripheral blood circRTN4 alleviated renal damage and suppressed FN and CyclinD1 expression in LN mice. **a** RT-qPCR for circRTN4 in exosomes of peripheral blood of BALB/c mice. **b** Twenty-four-hour proteinuria levels were detected by ELISA. **c**, **d** Level of BUN and Scr in LN mice. **e** IF staining quantitative analysis of CD68 in glomeruli of BALB/c mice. **f** H&E staining of kidney and analysis of cell number of glomeruli. **g** PAS staining and semiquantitative analysis of mesangial expansion and extracellular matrix deposition. **h** Sirius red staining and semiquantitative analysis of collagen in glomerular. **i** Western blot analysis and quantitative analysis (below) of FN protein levels in the renal cortex. **j** IF staining and quantitative analysis of FN protein levels in the glomeruli of BALB/c mice. **k** IHC assays and quantitative analysis of CyclinD1 levels in the glomeruli of BALB/c mice. **l** Western blot analysis of CyclinD1 protein levels in the renal cortex. *n* = 6 for each group. ^*^P < 0.05, ^**^P < 0.01 and ^***^P < 0.001, One-way ANOVA followed by Bonferroni’s test. Data are presented as means ± SEM.

Our pre-test had confirmed that the level of circRTN4 in PBMCs of mice could be effectively knocked down by AAV through tail vein injection (Supplementary Fig. S2a), but the virus could almost not infect glomerulus cells, only infects the renal tubule cells and renal interstitial (Supplementary Fig. S2b). To further confirm our hypothesis, two LN model mice were administered circRTN4-shRNA-AAV or NC-AAV by tail vein injection. As illustrated in Fig. 7a, the expression of circRTN4 in exosomes from the serum of mice was notably increased in BALB/c + P mice by RT‐qPCR, which could be suppressed by CircRTN4-shRNA-AAV. In addition, downregulation of circRTN4 alleviated proteinuria (Fig. 7b) and blood urea nitrogen (BUN) levels in the BALB/c + P + circRTN4-R group (Fig. 7c), while serum creatinine (Scr) remained unchanged (Fig. 7d). Moreover, the knockdown of circRTN4 alleviated macrophage infiltration (Fig. 7e) and relieved the pathological changes of the kidneys as shown in Fig. 7f–h by H&E, PAS and Sirius Red staining. Furthermore, the FN protein expression in glomerular cells was notably decreased in BALB/c + P + circRTN4-V group compared with that in BALB/c + P group (Fig. 7i, j). Consistently, the expression of CyclinD1 showed a similar result (Fig. 7k, l). Similar results were shown in MRL/lpr mice (Supplementary Fig. S2c–i). Taken together, peripheral blood-circRTN4 was contributed to the renal damage by promoting macrophage infiltration, mesangial cell proliferation, and ECM deposition in LN mice.

## Discussion

We report here that circRTN4 plays a crucial role in LN progression by regulating the miR-513a-5p/FN axis. Moreover, monocyte could participate in mesangial cell dysfunction in exosome dependent manner.

Our previous study has proved that mesangial cell dysfunction plays an important role in LN progression^22^. Meanwhile, expanding data have demonstrated that infiltrating monocytes and macrophages are associated with renal injury in both mice and human^23,24^. Consistent with these reports, we confirmed monocyte infiltration in renal tissues of LN. In this study, the results revealed that monocytes could aggravate mesangial cell dysfunction through exosomes in LN. Thus, we were eager to know the specific mechanism underlying this intracellular communication.

Recently, research on circRNAs has mainly focused on oncology, and they have been reported to play key roles in tumor processes^25–27^. Given that circRNAs are highly abundant and specifically expressed in tissue^15,16^, they may be involved in the pathology of multiple glomerulonephritis. Hu et al.^28^ confirmed that circRNA-15698 is highly expressed in both DN mice and mouse mesangial cells and can aggravate the extracellular matrix of DN. Ouyang et al.^29^ reported that plasma circRNA_002453 levels were upregulated in LN patients and positively correlated with 24-h proteinuria. In contrast, the expression of hsa_circ_0123190 was significantly decreased in the renal tissues of patients with LN^30^. Nevertheless, the specific mechanism of circRNAs in LN remains ambiguous.

In this study, we found that circRNA-0054595, named circRTN4, was significantly increased in mesangial cells and exosomes derived from monocytes of LN. This finding suggested that circRTN4 may be systematically involved in the progression of LN. Clinically, we also found that circRTN4 was upregulated in renal tissues, peripheral blood mononuclear cells (PBMCs) and exosomes of LN patients. Recent studies have shown that circRNAs can also be released in the form of exosomes and transported to responding cells, through which they affect the physiological behavior of recipient cells or mediate pathological injury. Exosome biology heralds the future arena of disease management, especially in immunotherapy, RNA therapy and stem cell therapy^31–33^. Wang et al. suggested that circRNA-002178 could be delivered into CD8 + T cells to induce PD1 expression via exosomes in lung adenocarcinoma^34^. Additionally, circ_DLGAP4 significantly promoted the proliferation and fibrosis of MC cells in diabetic kidney disease^35^. Is there a connection between circRTN4 in mesangial cells and monocytes? Subsequent studies have identified that highly expressed circRTN4 in exosomes derived from THP1 cells could effectively upregulate the level of circRTN4 in mesangial cells, which could be partially reversed by knockdown of circRTN4 or inhibition of exosome release in monocytes. These results suggested that upregulated circRTN4 in monocytes may be packaged in exosomes and internalized by glomerular mesangial cells, which results in a peak in circRTN4 levels in mesangial cells, accompanied by the excessive synthesis and secretion of FN. Our study further confirmed the existence of this novel method of communication between immune cells and glomerular cells, which expands research on the potential regulatory functions of circRNAs in the pathological processes of LN.

It is widely believed that proliferative mesangial cells can secrete extracellular matrix, which is one of the major pathogenic events in the progression of LN. Further research showed that high circRTN4 expression notably promoted mesangial cell proliferation by affecting the cell cycle. However, downregulation of circRTN4 suppressed the expression of FN. Our previous study also demonstrated that fibrotic proteins, such as FN and ColIV, played an important role in the ECM of mesangial cells in LN^22^. It has been well demonstrated that a large proportion of circRNAs are in the cytoplasm, and the most crucial mechanism through which they perform regulatory functions is miRNA sponging^15,16,36^. Herein, we identified renal circRTN4 to be distributed mainly in the cytoplasm of mesangial cells. Its regulatory potential in regulating FN expression depended on directly interacting with miR-513a-5p. Most importantly, the use of AAV to suppress kidney circRTN4 caused pronounced inhibition of ECM deposition in not only a pristane-induced mouse model of LN but also in MRL/lpr mice, a classic lupus mouse. Additionally, we administered AAV via tail vein injection, aiming to knock down the expression of circRTN4 in PBMCs. These mice also exhibited much less cell proliferation and ECM deposition in glomeruli, which, from another perspective, confirmed the communication between monocytes and mesangial cells in LNs. We conducted the same experiments in vivo using MRL/lpr mice, and the results are shown in the supplementary figure. However, the specific mechanism by which circRTN4 regulates proliferation needs further investigation.

In summary, our research demonstrated that the circRTN4/miR-513a-5p/FN axis played a critical pathological role in mesangial cell dysfunction. Exosomal circRTN4 mediated the cross-talk between monocytes and HRMCs, serving as an effective biomarker for LN detection and a novel therapeutic target.

## Supplementary information

Supplemental Material

## Data Availability

The data that support the findings of this study are available from the corresponding author upon reasonable request.

## References

[1] Javinani, A., Ashraf-Ganjouei, A., Aslani, S., Jamshidi, A. & Mahmoudi, M. Exploring the etiopathogenesis of systemic lupus erythematosus: a genetic perspective. *Immunogenetics* **71**, 283–297 (2019). 10.1007/s00251-019-01103-2 30671674

[2] Parikh, S. V., Almaani, S., Brodsky, S. & Rovin, B. H. Update on lupus nephritis: core curriculum. *Am. J. Kidney Dis.* **76**, 265–281 (2020). 10.1053/j.ajkd.2019.10.017 32220510

[3] Wright, R. D., Dimou, P., Northey, S. J. & Beresford, M. W. Mesangial cells are key contributors to the fibrotic damage seen in the lupus nephritis glomerulus. *J. Inflamm.* **16**, 22–36 (2019). 10.1186/s12950-019-0227-x PMC6857320 31807119

[4] Lv, L. L., Feng, Y., Wu, M., Wang, B. & Liu B. C. Exosomal miRNA-19b-3p of tubular epithelial cells promotes m1 macrophage activation in kidney injury. *Cell Death Differ.* **27**, 210–226 (2020). 10.1038/s41418-019-0349-y PMC7206053 31097789

[5] Meera, R., Jürgen, S., Sudha, V., Chandra, M., Jay, S. F. & Chaim, P. Phoenix from the flames: Rediscovering the role of the CD40-CD40L pathway in systemic lupus erythematosus and lupus nephritis. *Autoimmun Rev.* **19**, 102668 (2020). 10.1016/j.autrev.2020.102668 32942031

[6] Liu, L. et al. Integrative informatics analysis of transcriptome and identification of interacted genes in the glomeruli and tubules in CKD. *Front. Med.* **7**, 615306 (2021). 10.3389/fmed.2020.615306 PMC7906987 33644086

[7] Liu, Y. Cellular and molecular mechanisms of renal fibrosis. *Nat. Rev. Nephrol.* **7**, 684–696 (2011). 10.1038/nrneph.2011.149 PMC4520424 22009250

[8] Li, Y., Lee, P. Y. & Reeves, W. H. Monocyte and macrophage abnormalities in systemic lupus erythematosus. *Arch. Immunol. Ther. Exp.* **58**, 355–364 (2010). 10.1007/s00005-010-0093-y PMC3785254 20676786

[9] Wang, R., Zhao, H., Liu, Y., Li, Y. & Cai, J. Macrophage colony-stimulating factor could evaluate both disease activity and renal involvement in systemic lupus erythematosus. *Ann. Palliat.* **10**, 2098–2107 (2021). 10.21037/apm-20-2607 33549023

[10] Noronha, I. L., Kruger, C., Andrassy, K., Ritz, E. & Waldherr, R. In situ production of TNF-alpha, IL-1 beta and IL-2R in ANCA-positive glomerulonephritis. *Kidney Int.* **43**, 682–692 (1993). 10.1038/ki.1993.98 8455368

[11] Mathieu, M., Martin-Jaular, L., Lavieu, G. & Théry, C. Specificities of secretion and uptake of exosomes and other extracellular vesicles for cell-to-cell communication. *Nat. Cell Biol.* **21**, 9–17 (2019). 10.1038/s41556-018-0250-9 30602770

[12] Pathan, M. et al. Vesiclepedia 2019: A compendium of RNA, proteins, lipids and metabolites in extracellular vesicles. *Nucleic Acids Res.* **47**, D516–D519 (2019). 10.1093/nar/gky1029 PMC6323905 30395310

[13] Lasda, E. & Parker, R. Circular RNAs co-precipitate with extracellular vesicles: a possible mechanism for circRNA clearance. *PLoS One.* **11**, e0148407 (2016). 10.1371/journal.pone.0148407 PMC4743949 26848835

[14] xChen, L. L. & Yang, L. Regulation of circRNA biogenesis. *RNA Biol.* **12**:381–388 (2015). 10.1080/15476286.2015.1020271 PMC4615371 25746834

[15] Jeck, W. R. et al. Circular RNAs are abundant, conserved, and associated with ALU repeats. *RNA* **19**, 141–157 (2013). 10.1261/rna.035667.112 PMC3543092 23249747

[16] Memczak, S. et al. Circular RNAs are a large class of animal RNAs with regulatory potency. *Nature* **495**, 333–338 (2013). 10.1038/nature11928 23446348

[17] Ma, C. et al. Circular RNA hsa_circ_0004872 inhibits gastric cancer progression via the miR-224/Smad4/ADAR1 successive regulatory circuit. *Mol. Cancer.* **19**, 157–170 (2020). 10.1186/s12943-020-01268-5 PMC7654041 33172486

[18] Huang, Y., Zhang, Y., Jia, L., Liu, C. & Xu, F. Circular RNA ABCB10 promotes tumor progression and correlates with pejorative prognosis in clear cell renal cell carcinoma. *Int. J. Biol. Markers.* **34**, 176–183 (2019). 10.1177/1724600819842279 31106654

[19] Tian, S., Liu, X., Fan, Q., Ma, J., Yao, L. & Li, Y. Microarray expression and functional analysis of circular RNAs in the glomeruli of NZB/W F1 mice with lupus nephritis. *Exp. Ther. Med.* **18**, 2813–2824 (2019). 10.3892/etm.2019.7901 PMC6755417 31555374

[20] Halkom, A., Wu, H. & Lu, Q. The contribution of mouse models to our understanding of lupus. *Int. Rev. Immunol.* **39**(4),174–187 (2020). 10.1080/08830185.2020.1742712 32202964

[21] Cheng, Y., Wang, D., Wang, F., Liu, J. & Liang, M. Endogenous mir-204 protects the kidney against chronic injury in hypertension and diabetes. *J. Am. Soc. Nephrol.* **31**(7),1539–1554 (2020). 10.1681/ASN.2019101100 PMC7350998 32487559

[22] Feng, X. et al. HMGB1 protein promotes glomerular mesangial matrix deposition via TRL2 in lupus nephritis. *J. Cell Physiol.* **235**, 5111–5119 (2020). 10.1002/jcp.29379 31667864

[23] Kishimoto, D., Kirino, Y., Tamura, M., Takeno, M. & Nakajima, H. Dysregulated heme oxygenase-1low M2-like macrophages augment lupus nephritis via Bach1 induced by type I interferons. *Arthritis Res Ther.* **20**, 64–77 (2018). 10.1186/s13075-018-1568-1 PMC5894134 29636091

[24] Maria, N. I., Davidson, A. Renal macrophages and dendritic cells in SLE nephritis. *Curr. Rheumatol. Rep.* **19**, 81–90 (2017). 10.1007/s11926-017-0708-y 29119288

[25] Yu, J. et al. Circular RNA cSMARCA5 inhibits growth and metastasis in hepatocellular carcinoma. *J. Hepatol.* **68**, 1214–1227 (2018). 10.1016/j.jhep.2018.01.012 29378234

[26] Zhang, N. et al. Circular RNA circSATB2 promotes progression of non-small cell lung cancer cells. *Mol. Cancer.* **19**, 101–107 (2020). 10.1186/s12943-020-01221-6 PMC7268724 32493389

[27] Kristensen, L. S., Hansen, T. B., Venø, M. T. & Kjems, J. Circular RNAs in cancer: opportunities and challenges in the field. *Oncogene* **37**, 555–565 (2018). 10.1038/onc.2017.361 PMC5799710 28991235

[28] Hu, W., Han, Q., Zhao, L. & Wang, L. Circular RNA circRNA_15698 aggravates the extracellular matrix of diabetic nephropathy mesangial cells via miR-185/TGF-β1. *J. Cell Physiol.* **234**, 1469–1476 (2019). 10.1002/jcp.26959 30054916

[29] Ouyang, Q. et al. Using plasma circRNA_002453 as a novel biomarker in the diagnosis of lupus nephritis. *Mol. Immunol.* **101**, 531–538 (2018). 10.1016/j.molimm.2018.07.029 30172209

[30] Zhang, C. et al. Hsa_circ_0123190 acts as a competitive endogenous RNA to regulate APLNR expression by sponging hsa-miR-483-3p in lupus nephritis. *Arthritis Res. Ther.* **23**, 24–35 (2021). 10.1186/s13075-020-02404-8 PMC7805051 33436040

[31] Qu, M. et al. Dopamine-loaded blood exosomes targeted to brain for better treatment of Parkinson’s disease. *J. Control Rel.* **287**, 156–166 (2018). 10.1016/j.jconrel.2018.08.035 30165139

[32] Pullan, J. E. et al. Exosomes as drug carriers for cancer therapy. *Mol. Pharm.* **16**, 1789–1798 (2019). 10.1021/acs.molpharmaceut.9b00104 30951627

[33] Cheng, Q., Shi, X., Zhang, Y. Reprogramming exosomes for Immunotherapy. *Methods Mol. Biol.* **2097**, 197–209 (2020). 10.1007/978-1-0716-0203-4_12 PMC7461623 31776927

[34] Wang, J. et al. circRNA-002178 act as a ceRNA to promote PDL1/PD1 expression in lung adenocarcinoma. *Cell Death Dis.* **11**, 32–42 (2020). 10.1038/s41419-020-2230-9 PMC6965119 31949130

[35] Bai, S. et al. Exosomal circ_DLGAP4 promotes diabetic kidney disease progression by sponging miR-143 and targeting ERBB3/NF-kappaB/MMP-2 axis. *Cell Death Dis.* **11**, 1008–1020 (2020). 10.1038/s41419-020-03169-3 PMC7683700 33230102

[36] Kristensen, L. S. et al. The biogenesis, biology, and characterization of circular RNAs. *Nat. Rev. Genet.* **20**, 675–691 (2019). 10.1038/s41576-019-0158-7 31395983

