# Supplementary material for: CircRTN4 aggravates mesangial cell dysfunction by activating the miR-513a-5p/FN axis in lupus nephritis
Source: Lab Invest. 2022 May 6;102(9):966–78. doi: 10.1038/s41374-022-00788-6 (PMC9420678; doi:10.1038/s41374-022-00788-6)
Supplement: Supplementary file 1 — Supplemental Material [file 41374_2022_788_MOESM1_ESM.docx]

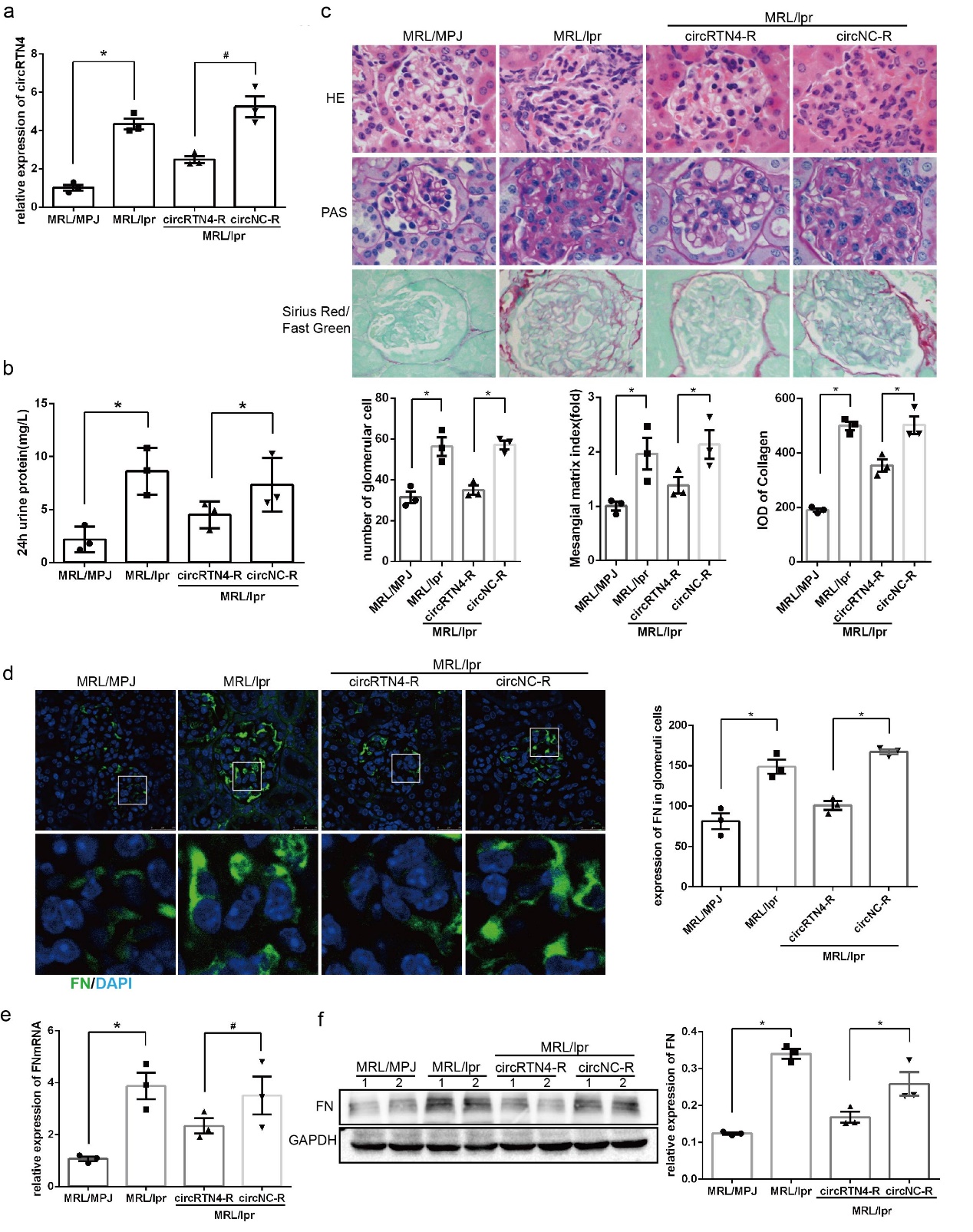


Supplementary figure1: Downregulation of circRTN4 alleviated renal damage and suppressed FN expression in MRL/lpr mice. (a) RT-qPCR assays showed the circRTN4 expression in renal cortex. (b) 24-h proteinuria levels were detected by ELISA. (c) HE, PAS and Sirius Red/Fast Green staining showed kidney histopathology and ECM deposition in LN mice. (d) IF staining for FN protein levels in the glomeruli. (e) RT-qPCR assays for FN mRNA levels in renal cortex (f) Western blot analysis and quantitative analysis (below) for FN protein levels in renal cortex. ^*^*P* <0.05, One-way ANOVA followed by Bonferroni’s test. Data are presented as means ± SEM.


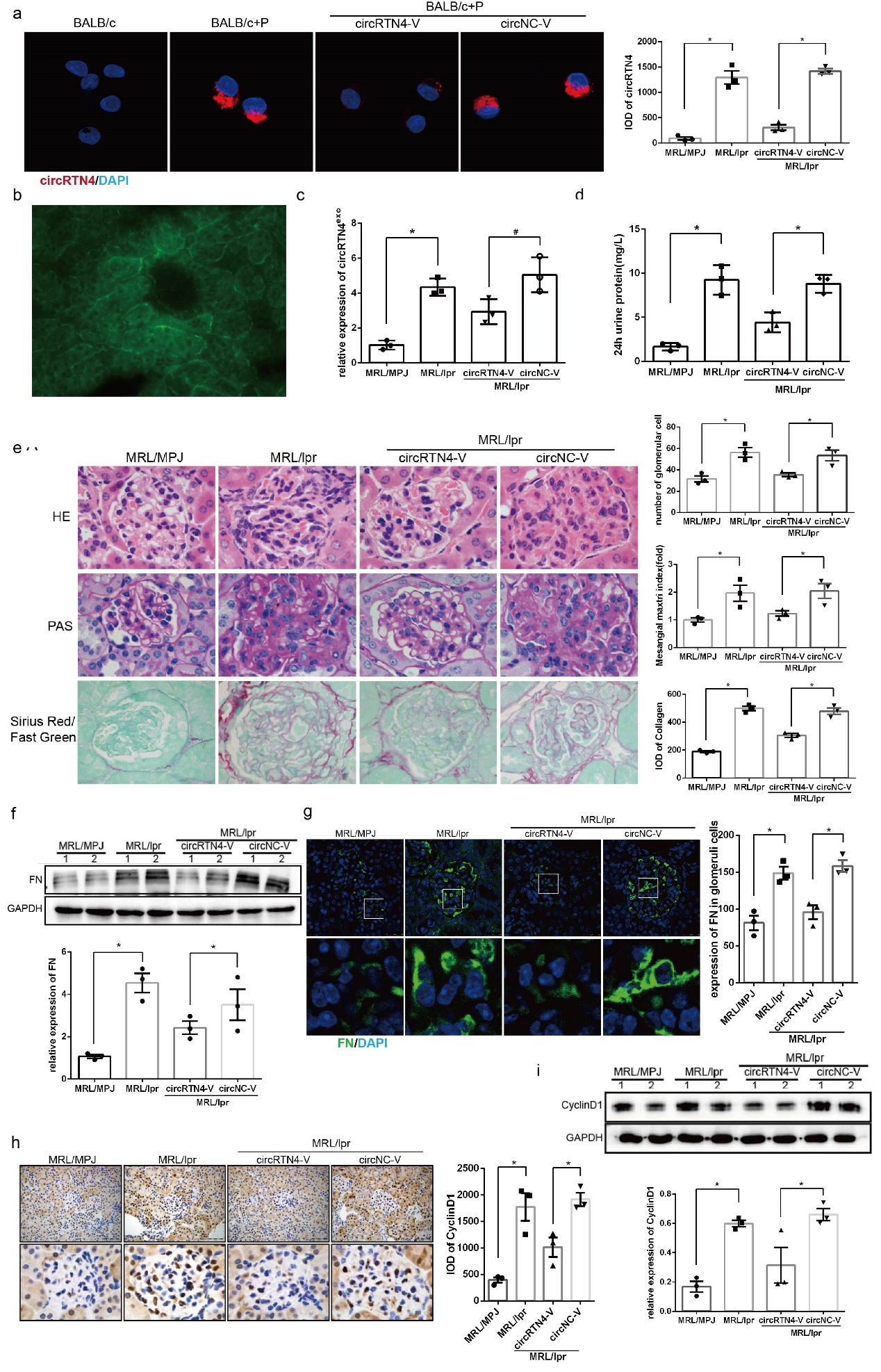


Supplementary figure2: Downregulation of peripheral blood circRTN4 alleviated renal damage and suppressed FN and CyclinD1 expression in MRL/lpr mice. (a) RNA FISH for circRTN4 in PBMCs of mice. (b) The expression of GFP protein in kidney of MRL/lpr mice after renally injected adenoviruses. (c) RT-qPCR for circRTN4 in exosomes of peripheral blood of MRL/lpr mice. (d) 24-h proteinuria levels were detected by ELISA. (e) HE, PAS and Sirius Red/Fast Green staining showed kidney histopathology and ECM deposition. (f) Western blot analysis and quantitative analysis (below) for FN protein levels in renal cortex. (g) IF staining for FN protein levels in kidney. (e) RT-qPCR assays for FN mRNA levels in renal cortex. (h, i) Western blot and IHC assays for cyclin D1 protein levels in t renal cortex. ^*^*P* <0.05, One-way ANOVA followed by Bonferroni’s test. Data are presented as means ± SEM.
